# Global trends in COVID-19 incidence and case fatality rates (2019–2023): a retrospective analysis

**DOI:** 10.3389/fpubh.2024.1355097

**Published:** 2024-07-29

**Authors:** Juan Du, Hong-mei Lang, Yan Ma, Ao-wen Chen, Yong-yi Qin, Xing-ping Zhang, Chang-quan Huang

**Affiliations:** ^1^Department of General Medicine, Chengdu Second People's Hospital, Chengdu, China; ^2^Department of Geriatrics, Chengdu Second People's Hospital, Chengdu, China

**Keywords:** COVID-19, infection rates, case-fatality rates, continents, income levels

## Abstract

**Objectives:**

Analyzing and comparing COVID-19 infection and case-fatality rates across different regions can help improve our response to future pandemics.

**Methods:**

We used public data from the WHO to calculate and compare the COVID-19 infection and case-fatality rates in different continents and income levels from 2019 to 2023.

**Results:**

The Global prevalence of COVID-19 increased from 0.011 to 0.098, while case fatality rates declined from 0.024 to 0.009. Europe reported the highest cumulative infection rate (0.326), with Africa showing the lowest (0.011). Conversely, Africa experienced the highest cumulative case fatality rates (0.020), with Oceania the lowest (0.002). Infection rates in Asia showed a steady increase in contrast to other continents which observed initial rises followed by decreases. A correlation between economic status and infection rates was identified; high-income countries had the highest cumulative infection rate (0.353) and lowest case fatality rate (0.006). Low-income countries showed low cumulative infection rates (0.006) but the highest case fatality rate (0.016). Initially, high and upper-middle-income countries experienced elevated initial infection and case fatality rates, which subsequently underwent significant reductions.

**Conclusions:**

COVID-19 rates varied significantly by continent and income level. Europe and the Americas faced surges in infections and low case fatality rates. In contrast, Africa experienced low infection rates and higher case fatality rates, with lower- and middle-income nations exceeding case fatality rates in high-income countries over time.

## 1 Introduction

Since its emergence at the end of 2019, the COVID-19 pandemic has rapidly spread globally. As of April 2023, COVID-19 has led to hundreds of millions of infections and millions of fatalities worldwide (1). Reducing the incidence and fatality rate of COVID-19 is a paramount public health concern. Recent advancements in vaccination and therapeutic interventions, along with the decrease in virulence of the COVID-19, have significantly and consistently reduced the mortality rate associated with this disease. However, the degree of the decline in COVID-19 mortality has shown regional and national variations.

The COVID-19 was first identified in China and quickly spread worldwide. During the initial phase of the epidemic, transmission occurred primarily in countries with higher economic status, particularly in Europe and the Americas. Subsequently, the outbreak spread to the African continent, leading to a second wave of infections that peaked in late 2020, affecting numerous countries across the region (2). Statistical estimates suggest that by 2021, approximately half of the world's countries had undergone all three waves of COVID-19 infections. Preliminary research suggests that during the initial wave of COVID-19 outbreaks, the transmission rate in countries with developing economies was lower than that in developed countries (3). However, the second wave of COVID-19 outbreaks in Africa has shown a concerning trend of increased severity compared to the first wave in the region. Numerous studies have explored the incidence and case fatality rates of COVID-19, revealing significant disparities across different trajectories. However, there remains a gap in longitudinal analyses that consider the entire span of the pandemic from 2019 to 2023 (4, 5). Given the dynamic nature of the COVID-19 pandemic and the evolution of variants, there is a critical need for retrospective analyses that span multiple years. This study aims to fill the existing research gap by providing a comprehensive overview of global COVID-19 trends, including infection and fatality rates, across different continents and income levels from 2019 to 2023.

## 2 Methods

### 2.1 Data source

Publicly available data from the World Health Organization (WHO) was utilized to investigate COVID-19 infections and deaths in various countries and regions from 2019 until April 2023. To ensure the reliability and completeness of the data, we included countries and territories that reported valid data on COVID-19 infections and deaths to WHO for each consecutive month from 2019 to 2023. Ultimately, 30 countries that provided incomplete or unpublished data were excluded, resulting in a total of 178 countries being analyzed. The countries included can be found in the Supplementary material.

We computed the COVID-19 infection rate and case fatality rate for each country. The COVID-19 infection rate was determined by calculating the cumulative number of confirmed COVID-19 cases as a percentage of the total population in the respective country or continent. The case fatality rate was calculated as the cumulative number of COVID-19 deaths divided by the cumulative number of confirmed COVID-19 cases. The calculation formulas for the infection rate and case fatality rate per 100,000 population can be expressed as follows: infection rate = (number of confirmed infections/total population) × 100,000. Case fatality rate = (number of COVID-19 deaths/number of confirmed infections) × 100,000. In order to compare differences among countries at various income levels, we followed the World Bank's classification into four categories: high-income, upper-middle-income, lower-middle-income, and low-income (6).

### 2.2 Statistical analysis

Data about the number of infections and deaths were collected for each time interval (2019–2020/6/1, 2020/7/1–2020/12/31, 2021/1/1–2021/6/30, 2021/7/1–2021/12/31, 2022/1/1–2022/6/30, 2022/7/1–2022/12/31, 2023/1/1–2023/4/1). Descriptive statistics were applied to quantify COVID-19 infection and mortality rates across different geographical areas and time periods. Rates per 100,000 population were calculated using the direct counts from the WHO for confirmed cases, deaths, and population figures. These computations facilitated the straightforward comparison of COVID-19's impact without necessitating inferential statistics. Python 3.12 and GraphPad Prism software were utilized for graphical representations of the calculated rates to enable clear and precise visualization of the trends across regions.

## 3 Results

### 3.1 General trends in the global prevalence and case-fatality rate of COVID-19

**Figure 1:**
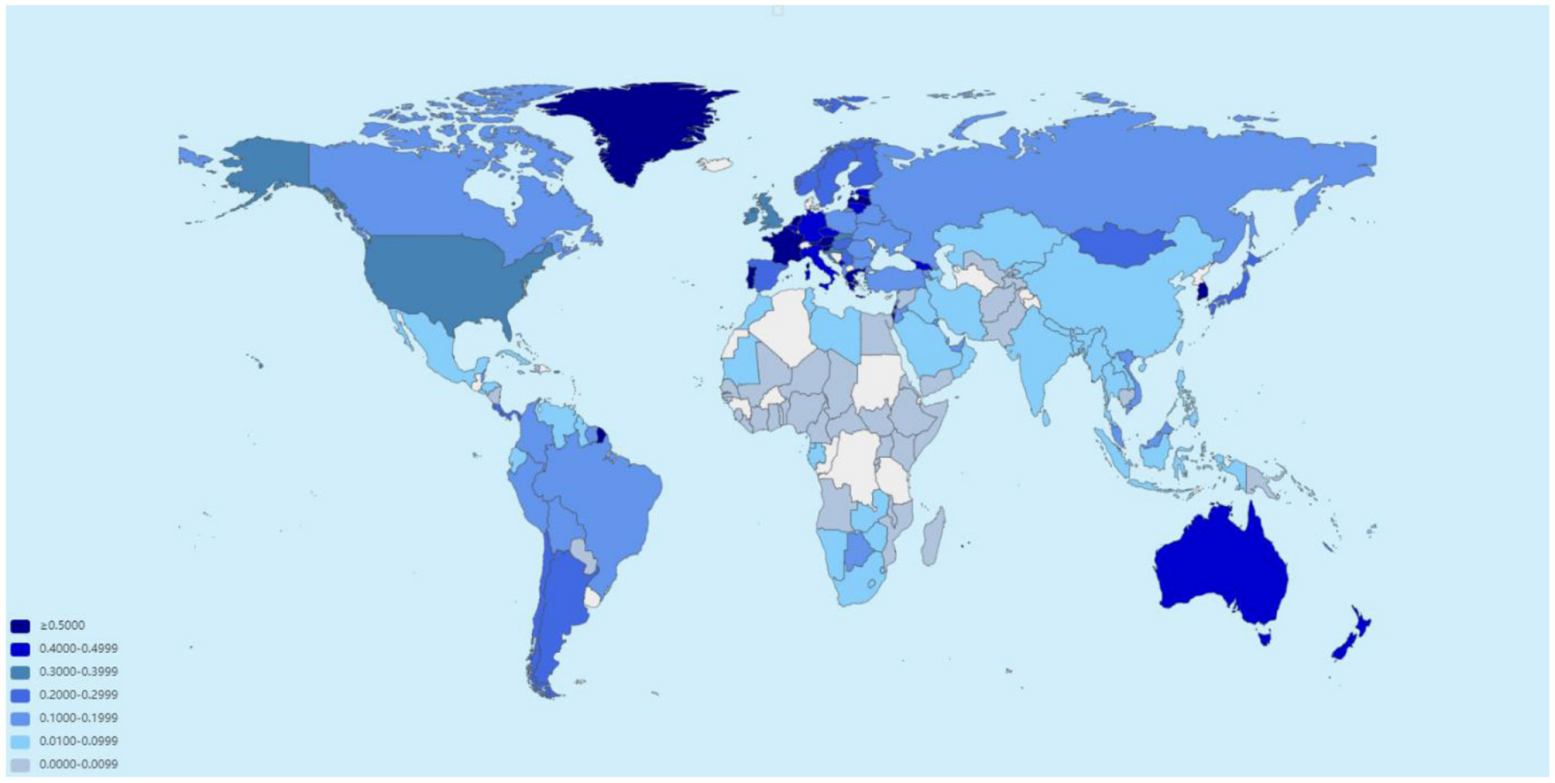
Estimated global incidence of COVID-19 as of April 2023 (data sourced from the World Health Organization).

**Figure 2:**
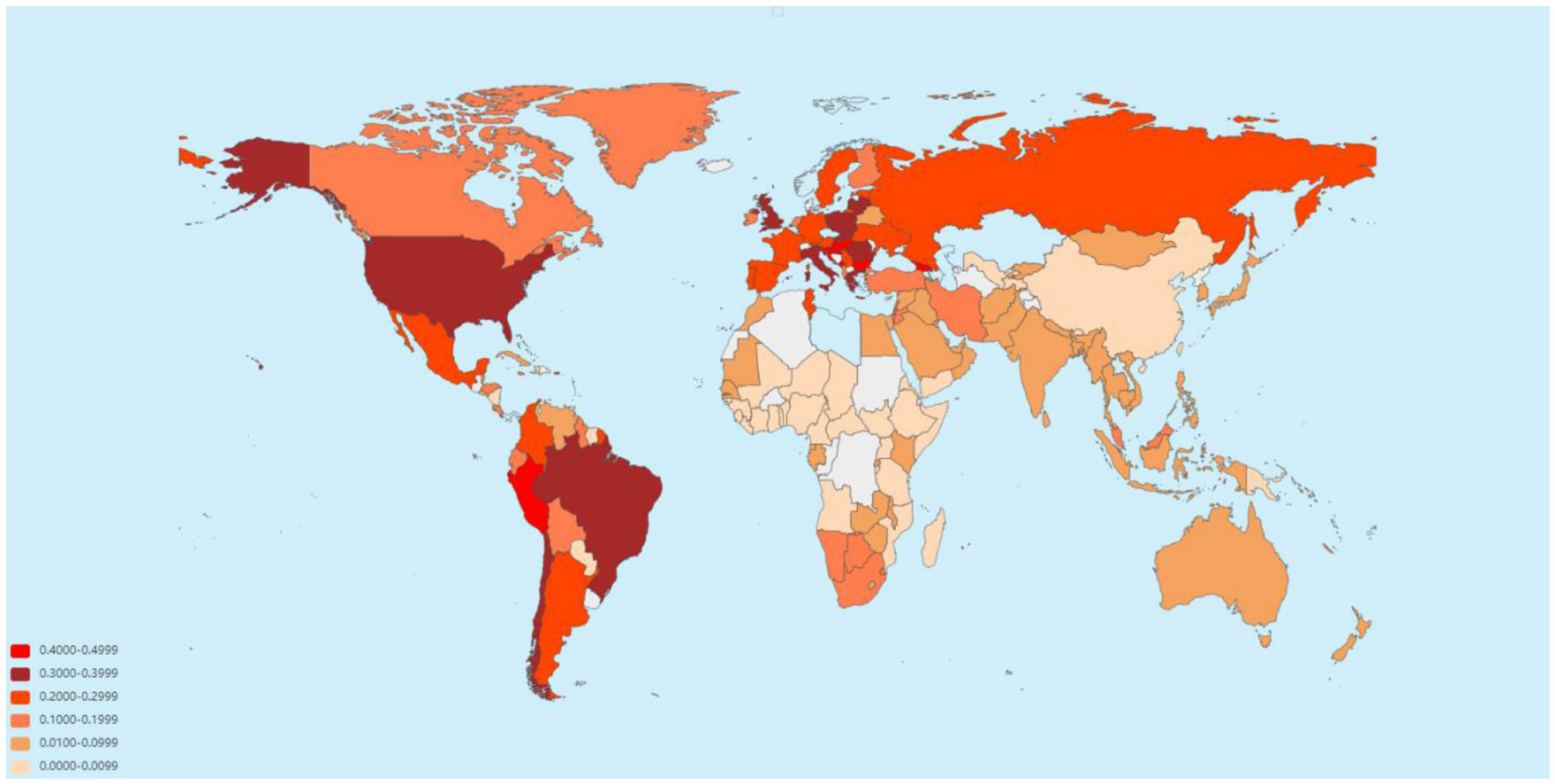
Estimated global case fatality rate of COVID-19 as of April 2023 (data sourced from the World Health Organization).

From the emergence of COVID-19 in late 2019 to April 2023, the number of reported COVID-19 infections totaled 762,133,867, with 6,811,298 associated deaths. Europe had the highest cumulative infection rate (0.326), while Africa had the lowest (0.011). Africa experienced the highest cumulative case-fatality rate (0.020), whereas Oceania saw the lowest (0.002). When categorized by income, high-income countries exhibited the highest cumulative infection rate (0.353) and the lowest case-fatality rate (0.006). In contrast, low-income countries had a lower cumulative infection rate (0.006) and the highest cumulative case-fatality rate (0.016; Figures 1, 2, Supplementary Tables 1, 2).

### 3.2 Temporal trends in infection rates across continents

**Figure 3:**
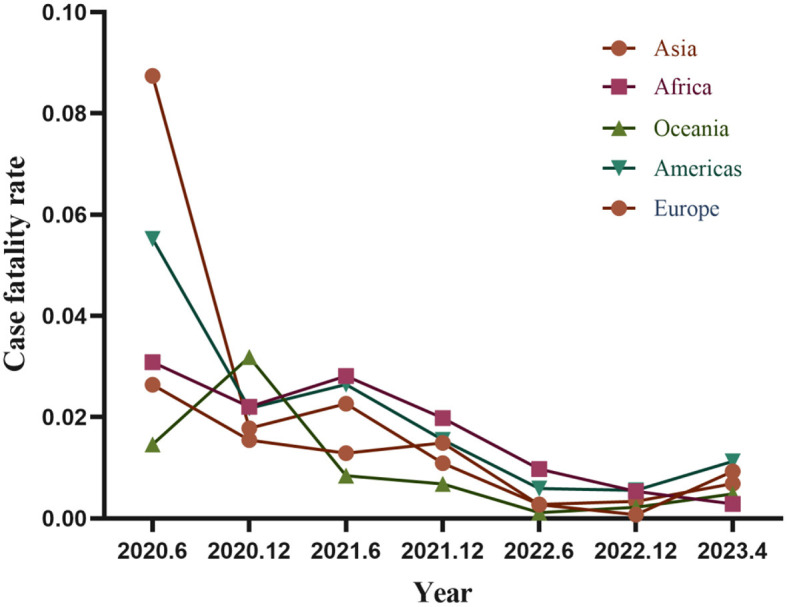
Trends in COVID-19 infection rates across continent (data sourced from the World Health Organization).

The COVID-19 infection rates in the Asian region showed an overall increasing trend from 2019 to April 2023. Conversely, the infection rates in the African region were comparatively lower, displaying an initial increase followed by a subsequent decrease. Similar patterns were observed in Europe, the Americas, and Oceania regions, mirroring those in Africa. Specifically, there were significant increases in infection rates from late 2021 to 2022, with Oceania experiencing minor increments. Since 2022, infection rates have gradually declined in Europe, the Americas, Oceania, and Africa. However, Asia experienced a persistent rise in infection rates, surpassing the other four continents by the first quarter of 2023 (Figure 3).

### 3.3 Temporal trends in case-fatality rates across continent

**Figure 4:**
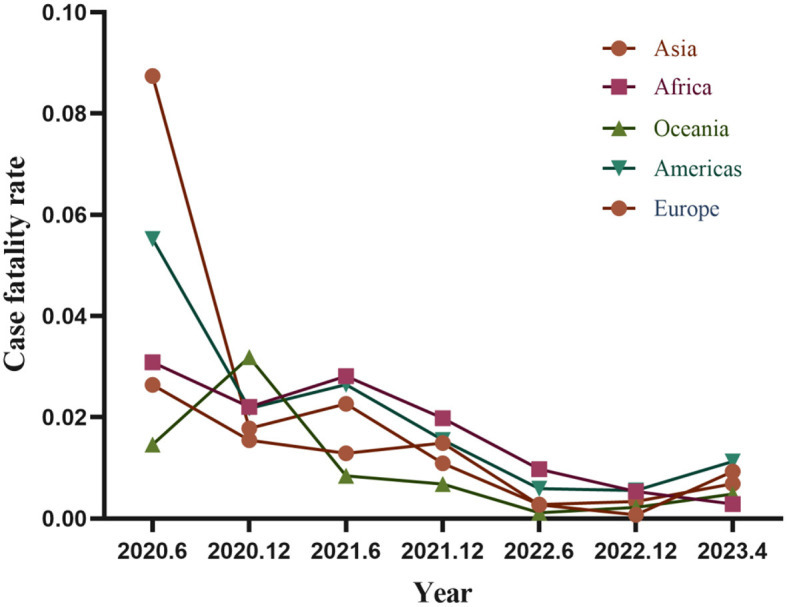
Temporal trends in case-fatality rates by continents.

All continents experienced a decline in both incidence and case-fatality rates, although with short-term fluctuations. Europe saw a notable decrease from 2019 to the end of 2020, with a slight increase in the first half of 2021 followed by a slow decline to lower lethality levels. Similarly, the Americas exhibited comparable temporal trends to Europe, but with higher case-fatality rates during the initial 6 months of the pandemic. Africa and Asia also showed declining case-fatality rates over time, with Africa experiencing a peak in mortality in the first half of 2021 and Asia seeing an upward trend in case-fatality rates compared to other continents from late 2022 to 2023. Oceania had the lowest case-fatality rates among continents, reaching a peaking in late 2020 but maintaining a low level afterward (Figure 4).

### 3.4 Temporal trends in infection rates in different income countries

There was a clear correlation between COVID-19 infection rates and the economic level of countries across different income categories. Over the past 3 years, high-income countries consistently had the highest infection rates, followed by upper-middle-income, lower-middle-income, and low-income countries with the lowest rates.

**Figure 5:**
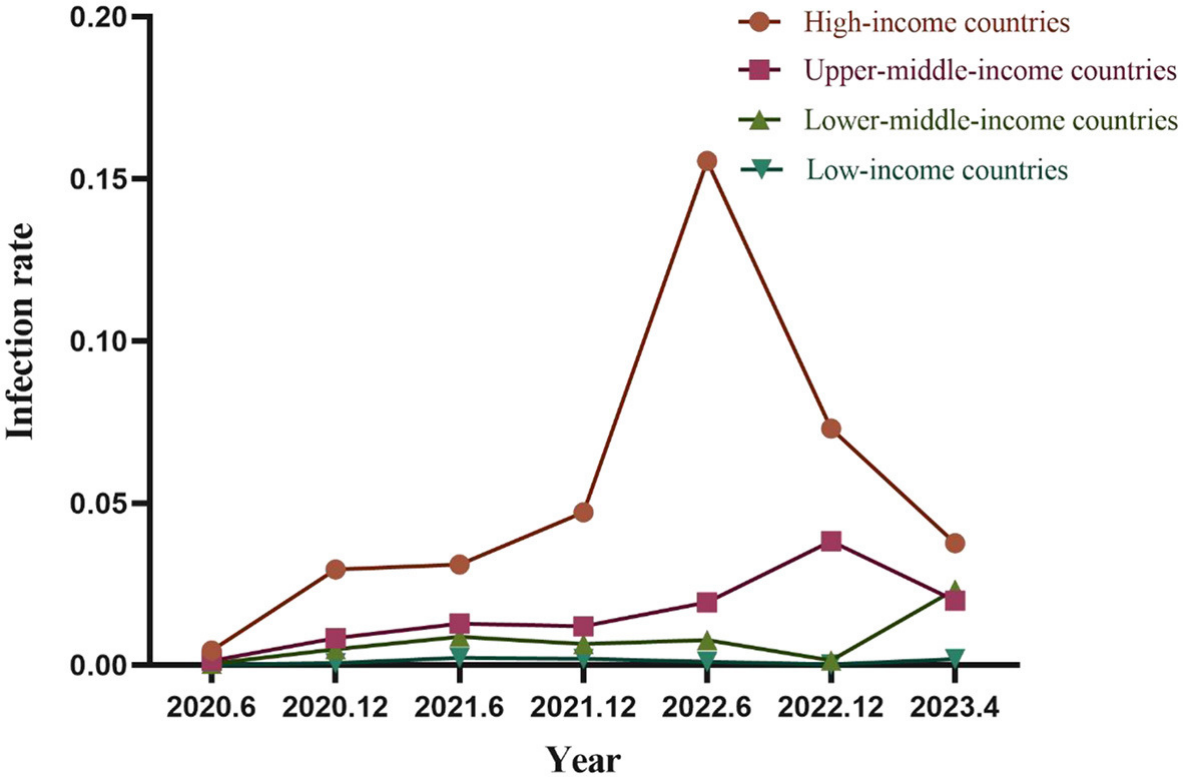
Temporal trends in infection rates in different income countries.

The trend in infection rates among high-income and upper-middle-income countries remained consistent, showing a continuous increase followed by a gradual decline in 2022. In contrast, infection rates in low-income and lower-middle-income countries remained low. However, low-income countries experienced a significant increase in infection rates in the first quarter of 2023, converging with the rates observed in upper-middle-income countries (Figure 5).

### 3.5 Time trends in case-fatality rate in different income countries

**Figure 6:**
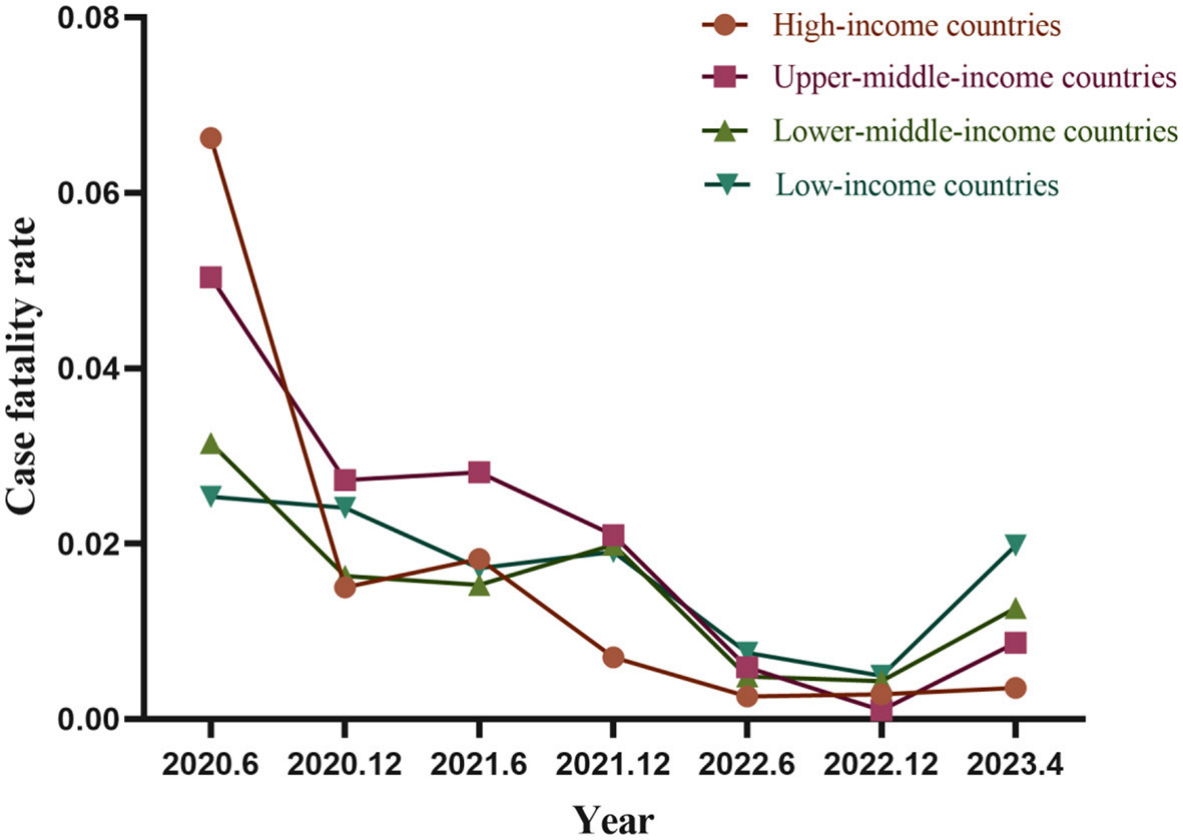
Temporal trends in case-fatality rate in different income countries.

Across countries with varying income levels, case-fatality rates generally showed a downward trend. During the first half of 2023, high-income and upper-middle-income countries exhibited higher rates than low-income and lower-middle-income countries, albeit with a more pronounced decline observed among high-income and upper-middle-income countries. Conversely, lower-middle-income and low-income countries experience a slower decline and intermittent fluctuations at different stages. Starting in 2022, lower-middle-income and low-income countries began to outpace high-income and upper-middle-income countries in terms of incidence and case-fatality rates. Nonetheless, except for high-income countries, all others experienced a slight upswing in the early years of 2023, despite still being well below the initial case fatality rates observed during the COVID-19 emergence (Figure 6).

## 4 Discussion

These findings indicate significant variations in COVID-19 infection and case fatality rates among continents as well as countries with different income levels. Building upon these results, we further analysis to examine the underlying factors contributing to these disparities.

### 4.1 Prevalence of COVID-19 infections across continents

In this study, we observed that Oceania initially had low infection rates, which then significantly increased in 2022. Several factors were identified as contributing to the initially low infection rates in Oceania. Firstly, Oceania's unique geography limited the movement of people between its islands and with other continents, thereby reducing the transmission channels of the virus (7). Secondly, Oceania is comprised of only 14 countries with low population densities. During the initial stages of COVID-19, Australia and New Zealand implemented strict lockdown measures, which were highly effective in controlling and preventing the spread of COVID-19. However, due to the unsustainable nature of prolonged lockdowns, both countries eventually lifted their isolation and containment policies in 2022 (8, 9). As a result, the number of infections in Oceania showed a significant upward trend in 2022 (10). We suspect that demographics and aging play significant roles in this trend. Countries in Europe exhibit higher population densities and a more significant proportion of an aging population than other regions, with Europe accounting for nine out of the 10 countries with the highest proportion of older adult individuals worldwide. Overall, policies and responses to COVID-19 outbreaks have varied across countries, with countries in Europe and the Americas adopting comparatively more lenient measures to prevent epidemic spread compared to other continents (11). Consequently, these regions have experience consistently high infection rates. The notable increase in COVID-19 infection rates in Asia during the first quarter of 2023 can be attributed to the gradual easing of strict control measures and the relaxation of movement restrictions in China since late 2022.

The persistence of low infection rates in Africa, on the other hand, may be attributed to the age structure of the continent. The report indicates that all the top 10 countries with the highest proportion of population in the 0–14 age group are located in Africa (12). Additionally, it is worth noting that testing for COVID-19 is relatively limited in African countries with lower economic incomes, which may result in an underestimation of mortality from COVID-19 in Africa (13).

Several studies have demonstrated a strong correlation between the prevalence of COVID-19 and environmental conditions. Factors such as the duration, temperatures, humidity, PM2.5, and sunlight exposure values (14). Specifically, some studies have indicated a positive relationship between PM2.5 and COVID-19 cases. Other studies suggest that higher ultraviolet (UV) radiation may inhibit airborne transmission of the virus. However, in outdoor environments, this effect may be influenced by factors such as humidity, temperature, and ventilation (15). Certain studies have found that vitamin D may reduce susceptibility to COVID-19 (16). For instance, one study correlated COVID-19 mortality data with mean 25(OH)D levels from multiple countries and reported similar observations. Another study noted that severe vitamin D deficiency is associated with comorbidities like chronic kidney disease (CKD), chronic obstructive pulmonary disease (COPD), and cerebrovascular disease (17), which increase the risk of COVID-19 infections. Sunlight is a major source of vitamin D for the body, especially in regions with extended daylight hours such as Oceania and Africa (18). Conversely, countries in Europe and America with shorter periods of sunlight have demonstrated greater susceptibility to COVID-19 infections. However, additional research is necessary to validate these hypotheses and unravel their underlying mechanisms.

### 4.2 Case-fatality rate trends across continents

At the onset of the epidemic, Africa had a higher COVID-19 mortality rate compared to Oceania. However, from 2021, a declining trend in COVID-19 mortality was observed in most continents except Africa, where the mortality rate remained high. As mentioned in the previous section, Oceania exhibited a low rate of COVID-19 infection during the initial year of the outbreak due to its favorable geographic location. The resilient healthcare system in Oceania also played a significant role in maintaining a low rate of COVID-19 mortality (19). Europe and North America are recognized for having the most advanced healthcare systems for COVID-19 with North American countries like the United States featuring advanced healthcare technologies and facilities. Additionally, Canada's universal healthcare system contributes significantly to the prevention and treatment of COVID-19. Asian countries exhibit substantial disparities in healthcare provision, with Japan and Singapore showcasing high rates of healthcare penetration (20). China has made notable progress in healthcare reform and development, although challenges persist regarding inconsistent service quality and resource allocation. African countries face healthcare system challenges, including inadequate resources and limited coverage (21). Undeniably, COVID-19 mortality is closely linked to a country's healthcare system. Accurately evaluating the influence of healthcare resources on COVID-19 mortality requires careful consideration of multiple factors.

In addition, effective vaccination is strongly associated with mortality reduction from COVID-19. Notably, there are significant variations in vaccination rates against COVID-19 across continents. The European and North American regions have demonstrated higher vaccination rates, resulting in a substantial number of deaths averted per vaccination administered within these geographic areas. In contrast, vaccination coverage varies significantly by country in Asian countries, and progress in Africa has been slow (22). Numerous studies have highlighted the potential benefits of vaccination, including the prevention of further mutations through decreased transmission of COVID-19, as well as the potential reduction in case fatality and overall mortality rates associated with COVID-19 (23).

The influence of climate, temperature, and ecology in different regions on COVID-19 mortality cannot be disregarded. A study revealed that cities with over 100,000 inhabitants experienced a higher COVID-19 mortality rate when there was a reduction in forest cover (24). It was also noted that countries in the Americas and Europe tend to have higher forest cover. Temperature has also been found to impact COVID-19 mortality rates. Higher mean monthly temperatures in European countries and temperate climates in the United States were associated with lower daily mortality rates. However, when considering the interaction between monthly temperature and vaccination rates, the effect of vaccination campaigns on mortality was more significant at lower temperatures than at higher temperatures (25). Multicenter research is required to elucidate further the relationship between the natural environment and COVID-19 mortality.

### 4.3 Prevalence of COVID-19 infections across economically diverse countries

Following the COVID-19 pandemic, nations across various income brackets have exhibited an overall escalation in their infection rates. High-income countries have consistently maintained a high infection rate, while low-income countries exhibit a significantly lower infection rate without an evident upward or downward trend. Within a few months following the spread of COVID-19, the epicenter of the pandemic quickly shifted from Asia to Europe and the Americas. The Asian countries, having experienced the SARS in 2003, responded more promptly to the current situation. In contrast, Western countries were slow in recognizing and responding to COVID-19, resulting in a steep increase in infections due to its high infectivity and delayed government interventions (26). Some low-income countries implemented strict lockdowns and restrictions during the early phases, resulting in limited international travel and population movement. These countries also have lower populations and a higher prevalence of outdoor living and working conditions than high-income countries (27). In addition, as previously mentioned high-income countries generally possess superior healthcare systems and information technology infrastructures, which allow them to collect, compile, and report infection data more accurately and promptly than low-income countries. Therefore, low-income countries may face difficulties in collecting complete data and experience time-consuming processes, leading to a potential underestimation of infection rates.

High-income countries also showed a clear upward trend in infection rates in mid-2022, possibly related to the emergence of omicron following the mutation of COVID-19 in late 2021. With the gradual liberalization of global vaccination measures, the strong transmissibility of omicron did not significantly reduce infection rates, despite the broader availability of vaccines in high-income and upper-middle-income countries.

In addition, aging demographics may play an essential role in the higher prevalence of COVID-19 because older people in low- and middle-income countries tend to be healthier on average than older people in wealthier countries. This difference may be because individuals with diseases in low- and middle-income countries face a higher risk of premature death at a young age (28). Aging demographics are often associated with weakened immune systems and the coexistence of multiple chronic diseases. In addition, high-income countries tend to experience more pronounced population aging due to their superior social security systems, higher life expectancy, and better overall health status (29). According to World Bank data, by 2021, the top 10 countries in terms of aging are predominantly high-income or upper-middle-income countries. This trend may contribute to the increased infection rates observed in high-income countries (30). A review suggests that countries with poorer sanitation and lower socioeconomic status may develop more robust innate immune systems during childhood due to frequent exposure to environmental microbes. This exposure may later reduce susceptibility to COVID-19 (31). In addition, another study found an association between higher rates of obesity and increased prevalence of COVID-19 in these 168 countries with available data. For every 1% increase in the prevalence of obesity, the incidence of COVID-19 cases increased by 6.6% (32). High-income countries, characterized by differences in diet and lifestyle, tend to have higher rates of obesity, which may contribute to a higher incidence of COVID-19 infection. Another study, which collected data from 61 countries worldwide, found a positive correlation between the number of COVID-19 cases and a country's GDP. The study highlighted countries with a higher ecological footprint and greater consumption had higher infection rates (33). Furthermore, high-income countries characterized by increased population mobility, higher levels of urbanization and greater population concentration also had correspondingly higher rates of COVID-19 infection.

### 4.4 Case-fatality rates in different income countries

A study published in 2022 reported a positive correlation between GDP and COVID-19 mortality, especially in wealthy countries (34). However, the results of our study indicate that as of April 2023, COVID-19 incidence was higher in high-income countries, while mortality rates were higher in low-income countries. This may be because high-income countries were among the first to be affected by virus transmission due to greater population mobility. As a result, during the early stages of the COVID-19 epidemic, high-income countries continued to experience elevated infection and mortality rates. However, they also had sufficient economic and medical resources to support significant reductions in infection rates and deaths.

According to a recent study, countries with higher hospital beds per 100,000 population showed lower COVID-19 incidence and mortality rates. Conversely, inadequate bed capacity and overcrowding in medical centers may contribute to increased mortality (35). Another study found that higher median population age, lower population density, and lower hospital bed availability were significantly associated with higher COVID-19 mortality rates (36). Notably, the median age of a country is highly correlated with its GDP per capita, with high-income countries typically having both a higher median age and a higher GDP (37). While these countries may have higher rates of COVID-19 infection and death, the overall mortality of patients with COVID-19 tends to be lower due to better medical and economic conditions. Globally, there is a significant disparity between the proportion of fully vaccinated population and COVID-19 mortality rates. As countries' income levels rise, vaccination rates increase and deaths per 1,000 cases decrease (38). However, following the emergence of the Omicron variant in December 2021, a significant proportion of the US population is fully vaccinated, highlighting the benefits of high vaccination rates in higher-income countries by showing lower case fatality rates for Omicron. However, by the end of 2022, only 76% of people aged 60 years and older had been fully vaccinated, well below the World Health Organization's target, particularly in low- and middle-income countries (39).

As noted above, several studies have suggested a possible association between obesity and increased susceptibility and mortality from COVID-19 (40). However, it is noteworthy that countries with a higher prevalence of obesity are often middle- and high-income countries with better healthcare resources. For example, countries such as Qatar, Germany and New Zealand, despite having obesity rates of over 25% in their adult populations, have significantly lower mortality rates despite high infection rates (41). Conversely, some South American countries with moderate levels of obesity still have higher mortality rates, mainly due to limited healthcare facilities. One study found that low BMI is associated with malnutrition, weakness and muscle wasting, all associated with an increased risk of COVID-19 mortality (42). In addition, low-income countries may face challenges such as wasting, weakened immune function and a higher prevalence of burdensome diseases such as diabetes and hypertension due to inadequate health systems and poor socioeconomic conditions. These factors may contribute to an increased risk of severe illness and death following COVID-19 infection.

Another study analyzing data from 81 countries found that countries with a higher proportion of urban population and higher rates of urbanization had, on average, the same or fewer COVID-19 deaths than less urbanized countries (43). Despite the faster spread of COVID-19 in urban areas, the number of deaths may be lower in highly urbanized countries due to economic development, trust in government and well-functioning health systems.

It is essential to acknowledge that in our discussion of case-fatality rates among different continents and income countries, Europe and the Americas consist predominantly of high-income countries. Conversely, out of the 50 countries in Africa, only Seychelles is classified as a high-income country. Therefore, we must recognize the interplay between region and income level.

In addressing the global disparity in COVID-19 infection and fatality rates, it is imperative to consider the impact of public health and social measures. Studies have shown that interventions like mask wearing, hand washing, and social distancing have varied efficacy across different regions, largely dependent on the rigor and consistency of their implementation (44). Furthermore, the economic responses to the pandemic have been significant, with policies ranging from health system investments to fiscal measures aimed at mitigating socio-economic impacts. For instance, OECD reports highlight the necessity of substantial investments in health systems to bolster resilience against public health emergencies, suggesting these investments are crucial components of broader economic stability strategies during crises (45). Additionally, geopolitical responses, including ongoing conflicts, travel restrictions, and changes to international trade policies, have influenced virus transmission rates and the economic interdependencies that shape global health outcomes (46, 47). These measures, while essential in controlling the spread of the virus, have also led to significant social and economic repercussions, which have been unevenly distributed and have exacerbated existing inequalities. Thus, understanding the full impact of these dynamics requires a multidimensional analysis that accounts for the complex interplay between public health initiatives and socio-economic factors.

## 5 Limitations

This study has several limitations that need to be acknowledged. Firstly, while the data utilized in this analysis comes from the World Health Organization (WHO), which employs rigorous methods to ensure accuracy and reliability, differences in COVID-19 reporting standards and statistics between countries and regions may still affect data comparability. These include variations in testing methods, standards, and reporting mechanisms. Despite these potential inconsistencies, the WHO continuously updates and verifies the data to reflect true trends as accurately as possible, thus maintaining the integrity of a globally recognized health data management system. Secondly, the analytical approach of this study was predominantly descriptive. We acknowledge that descriptive statistics do not permit causal inferences. However, they are invaluable for illustrating global and regional trends in COVID-19 infection and fatality rates, providing a foundational understanding that is essential for hypothesis generation and future analytical explorations. This approach was chosen due to the exploratory nature of the study and the limitations imposed by the available data. Moreover, the potential underreporting of COVID-19 cases and deaths, particularly in regions with limited testing capabilities and varying case definitions, may skew our understanding of the pandemic's true burden.

Despite these limitations, our study provides valuable insights into the global dynamics of the COVID-19 pandemic and underscores significant disparities in infection and mortality rates across different continents and income levels. Future research should not only aim to employ more comprehensive and precise data collection methods but also advance deeper and more analytical studies that consider demographic and other relevant factors. By extending beyond the mitigation of reporting biases, we can explore nuanced interpretations and foster more robust conclusions that will enhance our understanding of the pandemic's impact and support the development of more effective public health strategies and policies.

## 6 Conclusions

Overall, a decreasing trend in COVID-19 case-fatality rates was observed in different countries and regions, while infection rates showed an increasing trend. However, there were significant differences among different regions and income levels. Although mortality rates have notably decreased, especially in high-income countries, they remain high in many low-income countries. COVID-19 is likely to persist for a long time, requiring continued efforts to reduce infection rates and to conduct active research into prevention and treatment measures according to the geographical and economic conditions of different countries.

## Data availability statement

The datasets presented in this study can be found in online repositories. The names of the repository/repositories and accession number(s) can be found in the article/Supplementary material.

## Ethics statement

Ethical approval was not required for the study involving humans in accordance with the local legislation and institutional requirements. Written informed consent to participate in this study was not required from the participants or the participants' legal guardians/next of kin in accordance with the national legislation and the institutional requirements.

## Author contributions

JD: Writing – original draft. H-mL: Writing – review & editing. YM: Writing – review & editing. A-wC: Writing – review & editing. Y-yQ: Writing – review & editing. X-pZ: Writing – review & editing. C-qH: Writing – review & editing, Writing – original draft, Data curation.
