# Supplementary material for: Global trends in COVID-19 incidence and case fatality rates (2019–2023): a retrospective analysis
Source: Front Public Health. 2024 Jul 29;12:1355097. doi: 10.3389/fpubh.2024.1355097 (PMC11317462; doi:10.3389/fpubh.2024.1355097)
Supplement: Supplementary file 1 [file Data_Sheet_1.PDF]

Supplementary appendix

Table1 COVID-19 Incidence rates across 178 countries during periods globally

Country	2019-2020/6/30	2020/7/1-12/31	2021/1/1-6/30	2021/7/1-12/31	2022/1/1-6/30	2022/7/1-12/31	2023/1/1-4/1
Israel	0.0028	0.0445	0.0475	0.0614	0.3340	0.0459	0.4958
Singapore	0.0074	0.0025	0.0007	0.0366	0.1947	0.1294	0.2582
Saudi Arabia	0.0052	0.0049	0.0035	0.0020	0.0067	0.0019	0.0215
Japan	0.0001	0.0017	0.0045	0.0074	0.0601	0.1592	0.0329
Kuwait	0.0104	0.0240	0.0469	0.0143	0.0520	0.0042	0.1482
Qatar	0.0321	0.0164	0.0264	0.0096	0.0445	0.0360	0.1324
South Korea	0.0002	0.0009	0.0019	0.0092	0.3458	0.2092	0.3915
Guadeloupe	0.0005	0.0211	0.0223	0.0957	0.2825	0.0796	0.0052
Bahrain	0.0148	0.0373	0.0977	0.0093	0.1939	0.0399	0.3530
Oman	0.0074	0.0170	0.0260	0.0074	0.0161	0.0016	0.0738
United Arab Emirates	0.0048	0.0157	0.0423	0.0130	0.0183	0.0101	0.0951
Reunion Island	0.0006	0.0093	0.0244	0.0508	0.3819	0.0725	0.0067
New Zealand	0.0002	0.0001	0.0001	0.0023	0.2710	0.1471	0.0305
New Caledonia	0.0001	0.0001	0.0003	0.0470	0.1892	0.0560	0.0017
Australia	0.0003	0.0008	0.0001	0.0129	0.2851	0.1158	0.0132
Aruba	0.0010	0.0502	0.0548	0.0751	0.2065	0.2134	0.0024
Chile	0.0144	0.0172	0.0493	0.0130	0.1131	0.0547	0.0120
Virgin Islands	0.0008	0.0182	0.0174	0.0489	0.1113	0.0272	0.0103
Turks and Caicos Islands	0.0011	0.0216	0.0408	0.0225	0.0769	0.0068	0.0023
Saint Kitts and Nevis	0.0006	0.0000	0.0076	0.0460	0.0576	0.0100	0.0007

United States	0.0080	0.0506	0.0411	0.0605	0.0980	0.0392	0.0104
Curacao	0.0002	0.0261	0.0512	0.0494	0.1529	0.0074	0.0004
Canada	0.0027	0.0121	0.0222	0.0180	0.0476	0.0148	0.0037
Greenland	0.0002	0.0002	0.0004	0.0430	0.1662	0.0000	0.0000
Bahamas	0.0003	0.0194	0.0119	0.0297	0.0287	0.0039	0.0000
Gibraltar	0.0052	0.0499	0.0791	0.1293	0.3328	0.0309	0.0049
Jersey	0.0031	0.0242	0.0084	0.1607	0.3488	0.1203	0.0061
United Kingdom	0.0041	0.0333	0.0340	0.1250	0.1357	0.0089	0.0126
Italy	0.0040	0.0306	0.0361	0.0286	0.2066	0.1116	0.0087
Hungary	0.0004	0.0331	0.0505	0.0466	0.0699	0.0268	0.0014
Greece	0.0003	0.0130	0.0275	0.0724	0.2588	0.1689	0.0365
Spain	0.0055	0.0364	0.0401	0.0573	0.1333	0.0193	0.0067
Slovenia	0.0008	0.0581	0.0650	0.0995	0.2765	0.1295	0.0160
Slovakia	0.0003	0.0326	0.0388	0.0824	0.1748	0.0116	0.0012
San Marino	0.0210	0.0495	0.0802	0.0914	0.2868	0.1509	0.0196
Sweden	0.0066	0.0373	0.0629	0.0232	0.1170	0.0157	0.0021
Portugal	0.0041	0.0340	0.0472	0.0483	0.3738	0.0399	0.0021
Norway	0.0016	0.0073	0.0150	0.0479	0.1913	0.0052	0.0011
Isle of Man	0.0038	0.0005	0.0137	0.1530	0.2347	0.0261	0.0000
Liechtenstein	0.0022	0.0557	0.0237	0.0822	0.3021	0.0890	0.0040
Lithuania	0.0007	0.0537	0.0502	0.0919	0.2385	0.0477	0.0098
Latvia	0.0006	0.0215	0.0521	0.0751	0.3016	0.0747	0.0014
Croatia	0.0007	0.0506	0.0372	0.0861	0.1077	0.0286	0.0017
Czech Republic	0.0011	0.0663	0.0898	0.0773	0.1583	0.0339	0.0050
Netherlands	0.0029	0.0428	0.0522	0.0832	0.2943	0.0228	0.0024
Guernsey	0.0038	0.0007	0.0081	0.1047	0.2902	0.1055	0.0168

Finland	0.0013	0.0052	0.0108	0.0302	0.1602	0.0541	0.0024
Faroe Islands	0.0038	0.0086	0.0035	0.1022	0.5916	0.0000	0.0000
France	0.0024	0.0367	0.0473	0.0598	0.3174	0.1192	0.0079
Germany	0.0023	0.0184	0.0237	0.0410	0.2515	0.1088	0.0116
Denmark	0.0022	0.0255	0.0231	0.0837	0.4093	0.0375	0.0035
Belgium	0.0053	0.0501	0.0371	0.0874	0.1848	0.0372	0.0081
Austria	0.0016	0.0373	0.0318	0.0689	0.3500	0.1398	0.0368
Andorra	0.0110	0.1166	0.0518	0.1189	0.2667	0.0513	0.0023
Estonia	0.0015	0.0197	0.0781	0.0836	0.2583	0.0218	0.0033
Ireland	0.0051	0.0128	0.0366	0.0958	0.1689	0.0187	0.0031
China	0.0001	0.0000	0.0000	0.0000	0.0032	0.0569	0.0084
Jordan	0.0001	0.0284	0.0445	0.0304	0.0813	0.0148	0.1847
Iran	0.0026	0.0116	0.0230	0.0350	0.0122	0.0038	0.0847
Turkey	0.0023	0.0234	0.0378	0.0476	0.0660	0.0220	0.1771
Thailand	0.0000	0.0001	0.0036	0.0281	0.0328	0.0028	0.0647
Malaysia	0.0003	0.0031	0.0194	0.0608	0.0546	0.0138	0.1389
Lebanon	0.0003	0.0263	0.0547	0.0273	0.0574	0.0165	0.1678
Kazakhstan	0.0016	0.0006	0.0229	0.0309	0.0169	0.0051	0.0733
South Africa	0.0024	0.0148	0.0175	0.0246	0.0090	0.0009	0.0004
Namibia	0.0001	0.0091	0.0257	0.0220	0.0066	0.0016	0.0003
Mauritius	0.0003	0.0001	0.0013	0.0700	0.1106	0.0472	0.0044
Gabon	0.0023	0.0018	0.0090	0.0073	0.0027	0.0004	0.0000
Equatorial Guinea	0.0024	0.0012	0.0047	0.0034	0.0016	0.0007	0.0000
Botswana	0.0001	0.0060	0.0235	0.0620	0.0417	0.0019	0.0007
Jamaica	0.0002	0.0041	0.0125	0.0146	0.0165	0.0034	0.0006
Saint Vincent	0.0003	0.0008	0.0189	0.0335	0.0278	0.0044	0.0006

Saint Lucia	0.0001	0.0012	0.0171	0.0284	0.0469	0.0096	0.0010
Mexico	0.0021	0.0093	0.0080	0.0114	0.0156	0.0091	0.0021
Peru	0.0083	0.0218	0.0309	0.0071	0.0397	0.0248	0.0011
Grenada	0.0002	0.0009	0.0003	0.0521	0.1076	0.0124	0.0001
Costa Rica	0.0006	0.0317	0.0382	0.0400	0.0775	0.0377	0.0115
Colombia	0.0018	0.0296	0.0500	0.0185	0.0197	0.0038	0.0003
French Guiana	0.0121	0.0297	0.0460	0.0672	0.1234	0.0351	0.0005
Puerto Rico	0.0007	0.0279	0.0208	0.0516	0.1731	0.0973	0.0197
Brazil	0.0063	0.0289	0.0506	0.0177	0.0463	0.0189	0.0046
Panama	0.0072	0.0466	0.0368	0.0203	0.0964	0.0239	0.0023
Barbados	0.0003	0.0010	0.0129	0.0842	0.1933	0.0748	0.0045
Antigua and Barbuda	0.0007	0.0009	0.0111	0.0305	0.0436	0.0048	0.0000
Argentina	0.0017	0.0347	0.0630	0.0307	0.0738	0.0114	0.0034
Serbia	0.0016	0.0370	0.0440	0.0670	0.0844	0.0481	0.0084
Romania	0.0014	0.0316	0.0238	0.0381	0.0584	0.0204	0.0031
Montenegro	0.0008	0.0751	0.0837	0.1085	0.1141	0.0712	0.0073
Russia	0.0044	0.0172	0.0162	0.0342	0.0544	0.0231	0.0058
Poland	0.0009	0.0335	0.0419	0.0325	0.0505	0.0094	0.0034
Bulgaria	0.0007	0.0286	0.0322	0.0470	0.0623	0.0175	0.0012
Belarus	0.0065	0.0138	0.0237	0.0298	0.0302	0.0012	0.0000
Vietnam	0.0000	0.0000	0.0002	0.0175	0.0912	0.0079	0.1089
Indonesia	0.0002	0.0025	0.0052	0.0147	-0.0006	0.0023	0.0220
India	0.0004	0.0069	0.0143	0.0032	0.0061	0.0009	0.0310
Iraq	0.0011	0.0131	0.0178	0.0180	0.0061	0.0028	0.0561
Armenia	0.0086	0.0451	0.0221	0.0404	0.0263	0.0075	0.1434
Uzbekistan	0.0002	0.0020	0.0010	0.0026	0.0012	0.0003	0.0071

Sri Lanka	0.0001	0.0019	0.0100	0.0152	0.0036	0.0004	0.0308
Myanmar	0.0000	0.0023	0.0006	0.0067	0.0015	0.0004	0.0111
Bangladesh	0.0009	0.0022	0.0024	0.0040	0.0023	0.0004	0.0118
Mongolia	0.0001	0.0003	0.0347	0.1711	0.0700	0.0233	0.2762
Laos	0.0000	0.0000	0.0003	0.0148	0.0132	0.0010	0.0282
Cambodia	0.0000	0.0000	0.0029	0.0041	0.0009	0.0001	0.0080
Kyrgyzstan	0.0010	0.0111	0.0066	0.0089	0.0024	0.0008	0.0300
Georgia	0.0002	0.0570	0.0349	0.1479	0.1780	0.0376	0.4247
Philippines	0.0003	0.0039	0.0084	0.0128	0.0076	0.0032	0.0332
Bhutan	0.0001	0.0008	0.0018	0.0007	0.0727	0.0035	0.0762
Palestine	0.0005	0.0285	0.0355	0.0240	0.0356	0.0082	0.1242
Pakistan	0.0009	0.0012	0.0021	0.0015	0.0011	0.0002	0.0067
Azerbaijan	0.0016	0.0195	0.0116	0.0273	0.0172	0.0032	0.0775
Tunisia	0.0001	0.0113	0.0231	0.0259	0.0272	0.0079	0.0004
Senegal	0.0004	0.0007	0.0018	0.0018	0.0007	0.0001	0.0000
Nigeria	0.0001	0.0003	0.0005	0.0003	0.0001	0.0000	0.0000
Morocco	0.0003	0.0113	0.0028	0.0114	0.0067	0.0016	0.0000
Libya	0.0001	0.0141	0.0134	0.0278	0.0162	0.0007	0.0000
Lesotho	0.0000	0.0012	0.0041	0.0084	0.0020	0.0002	0.0000
Kenya	0.0001	0.0016	0.0017	0.0020	0.0007	0.0002	0.0000
Cote d'Ivoire	0.0003	0.0005	0.0013	0.0007	0.0006	0.0002	0.0000
Cameroon	0.0005	0.0005	0.0024	0.0010	0.0004	0.0001	0.0000
Ghana	0.0006	0.0011	0.0018	0.0016	0.0006	0.0001	0.0000
Republic of Guinea	0.0004	0.0006	0.0011	0.0006	0.0004	0.0001	0.0000
Cape Verde	0.0021	0.0188	0.0386	0.0132	0.0364	0.0047	0.0001
Benin	0.0001	0.0002	0.0005	0.0013	0.0002	0.0001	0.0000

Angola	0.0004	0.0025	0.0015	0.0023	0.0014	0.0001	0.0000
Egypt	0.0006	0.0007	0.0020	0.0010	0.0012	0.0000	0.0000
Fiji	0.0000	0.0000	0.0048	0.0545	0.0130	0.0033	0.0002
Papua New Guinea	0.0000	0.0001	0.0018	0.0021	0.0009	0.0002	0.0000
Suriname	0.0008	0.0094	0.0256	0.0515	0.0484	0.0012	0.0015
El Salvador	0.0009	0.0061	0.0050	0.0066	0.0073	0.0049	0.0000
Nicaragua	0.0003	0.0004	0.0003	0.0010	0.0002	0.0001	0.0000
Honduras	0.0018	0.0101	0.0137	0.0117	0.0047	0.0038	0.0006
Guyana	0.0003	0.0077	0.0171	0.0244	0.0353	0.0061	0.0013
Cuba	0.0002	0.0008	0.0156	0.0688	0.0124	0.0005	0.0001
Ecuador	0.0031	0.0086	0.0136	0.0049	0.0200	0.0076	0.0008
Belize	0.0001	0.0258	0.0063	0.0460	0.0768	0.0155	0.0020
Bolivia	0.0026	0.0105	0.0233	0.0137	0.0271	0.0195	0.0033
Paraguay	0.0000	0.0003	0.0014	0.0005	0.0003	0.0001	0.0000
Ukraine	0.0010	0.0234	0.0273	0.0331	0.0312	0.0079	0.0025
Georgia	0.0002	0.0570	0.0349	0.1432	0.1827	0.0376	0.0066
Bosnia and Herzegovina	0.0013	0.0328	0.0289	0.0263	0.0271	0.0069	0.0004
Azerbaijan	0.0016	0.0195	0.0116	0.0273	0.0172	0.0032	0.0003
Albania	0.0008	0.0191	0.0263	0.0266	0.0245	0.0187	0.0004
Yemen	0.0000	0.0000	0.0002	0.0001	0.0001	0.0000	0.0004
Syria	0.0000	0.0006	0.0007	0.0013	0.0003	0.0001	0.0029
Tajikistan	0.0006	0.0008	0.0000	0.0004	0.0000	0.0000	0.0018
Nepal	0.0004	0.0004	0.0204	0.0063	0.0050	0.0007	0.0326
Afghanistan	0.0008	0.0005	0.0016	0.0010	0.0006	0.0006	0.0046
Central African	0.0009	0.0003	0.0019	0.0003	0.0004	0.0001	0.0000

Republic							
Chad	0.0001	0.0001	0.0002	0.0000	0.0001	0.0000	0.0000
Zambia	0.0001	0.0010	0.0069	0.0050	0.0039	0.0005	0.0005
Uganda	0.0000	0.0007	0.0010	0.0013	0.0005	0.0001	0.0000
Tanzania	0.0000	0.0000	0.0000	0.0005	0.0001	0.0001	0.0000
Somalia	0.0002	0.0001	0.0008	0.0005	0.0002	0.0000	0.0000
Eswatini	0.0007	0.0070	0.0090	0.0392	0.0061	0.0008	0.0003
Sierra Leone	0.0002	0.0001	0.0005	0.0002	0.0001	0.0000	0.0000
Niger	0.0000	0.0001	0.0001	0.0001	0.0001	0.0000	0.0000
South Sudan	0.0002	0.0001	0.0014	-0.0002	0.0002	0.0001	0.0000
Mozambique	0.0000	0.0005	0.0018	0.0033	0.0013	0.0001	0.0001
Republic of the Congo	0.0012	0.0018	0.0053	0.0067	0.0021	0.0006	0.0001
Mauritania	0.0009	0.0020	0.0022	0.0042	0.0038	0.0008	0.0000
Mali	0.0001	0.0002	0.0004	0.0003	0.0005	0.0001	0.0000
Malawi	0.0001	0.0003	0.0015	0.0019	0.0006	0.0001	0.0000
Madagascar	0.0001	0.0005	0.0009	0.0003	0.0005	0.0001	0.0000
Rwanda	0.0001	0.0005	0.0023	0.0054	0.0014	0.0001	0.0000
Liberia	0.0001	0.0002	0.0006	0.0005	0.0002	0.0001	0.0000
Comoros	0.0002	0.0005	0.0038	0.0029	0.0018	0.0005	0.0005
Zimbabwe	0.0000	0.0009	0.0023	0.0107	0.0029	0.0003	0.0003
Guinea-Bissau	0.0008	0.0004	0.0016	0.0013	0.0010	0.0003	0.0002
Gambia	0.0000	0.0015	0.0009	0.0016	0.0007	0.0002	0.0000
Eritrea	0.0001	0.0003	0.0013	0.0006	0.0005	0.0001	0.0000
Togo	0.0001	0.0003	0.0013	0.0019	0.0008	0.0002	0.0000
Burundi	0.0000	0.0001	0.0004	0.0020	0.0010	0.0008	0.0001
Ethiopia	0.0000	0.0010	0.0013	0.0012	0.0006	0.0001	0.0000

Vanuatu	0.0000	0.0000	0.0000	0.0000	0.0352	0.0023	0.0000
Solomon Islands	0.0000	0.0000	0.0000	0.0000	0.0300	0.0042	0.0000
Venezuela	0.0002	0.0037	0.0054	0.0060	0.0028	0.0008	0.0001
Haiti	0.0005	0.0004	0.0008	0.0006	0.0005	0.0002	0.0000

Table 2. COVID-19 Case Fatality Rates across 178 countries during periods globally.

	2019-2020/6/30	2020/7/1-12/31	2021/1/1-6/30	2021/7/1-12/31	2022/1/1-6/30	2022/7/1-12/31	2023/1/1- 4/1
Israel	0.0133	0.0076	0.0073	0.0033	0.0009	0.0026	0.0026
Singapore	0.0006	0.0002	0.0017	0.0036	0.0005	0.0004	0.0009
Saudi Arabia	0.0086	0.0262	0.0129	0.0153	0.0014	0.0045	0.0122
Japan	0.0524	0.0115	0.0199	0.0039	0.0017	0.0013	0.0040
Kuwait	0.0077	0.0056	0.0050	0.0081	0.0004	0.0008	0.0040
Qatar	0.0012	0.0027	0.0044	0.0010	0.0005	0.0001	0.0017
South Korea	0.0220	0.0129	0.0115	0.0072	0.0011	0.0007	0.0013
Guadeloupe	0.0769	0.0167	0.0126	0.0147	0.0011	0.0014	0.0058
Bahrain	0.0032	0.0036	0.0059	0.0026	0.0003	0.0006	0.0024
Oman	0.0043	0.0148	0.0126	0.0319	0.0017	0.0000	0.0118
United Arab Emirates	0.0065	0.0022	0.0027	0.0027	0.0008	0.0003	0.0024
Reunion Island	0.0038	0.0047	0.0089	0.0037	0.0012	0.0014	0.0023
New Zealand	0.0187	0.0048	0.0017	0.0022	0.0011	0.0012	0.0022
New Caledonia	0.0000	0.0000	0.0000	0.0220	0.0006	0.0001	0.0000
Australia	0.0139	0.0394	0.0033	0.0045	0.0011	0.0024	0.0060
Aruba	0.0297	0.0087	0.0101	0.0094	0.0018	0.0110	0.0276
Chile	0.0202	0.0331	0.0169	0.0263	0.0089	0.0044	0.0057

Virgin Islands	0.0741	0.0088	0.0038	0.0114	0.0025	0.0031	0.0027
Turks and Caicos Islands	0.0244	0.0061	0.0077	0.0093	0.0034	0.0000	0.0233
Saint Kitts and Nevis	0.0000	0.0000	0.0074	0.0101	0.0048	0.0056	0.0000
United States	0.0482	0.0132	0.0181	0.0108	0.0058	0.0056	0.0105
Curacao	0.0417	0.0031	0.0137	0.0078	0.0037	0.0179	0.0290
Canada	0.0821	0.0147	0.0126	0.0058	0.0066	0.0132	0.0186
Greenland				0.0004	0.0021		
Bahamas	0.1058	0.0205	0.0159	0.0397	0.0087	0.0103	
Gibraltar	0.0000	0.0037	0.0344	0.0014	0.0004	0.0060	0.0064
Jersey	0.1020	0.0046	0.0324	0.0013	0.0012	0.0013	0.0247
United Kingdom	0.1975	0.0164	0.0263	0.0026	0.0026	0.0247	0.0072
Italy	0.1443	0.0211	0.0248	0.0056	0.0025	0.0025	0.0082
Hungary	0.1411	0.0281	0.0421	0.0205	0.0111	0.0072	0.0176
Greece	0.0563	0.0342	0.0279	0.0107	0.0036	0.0027	0.0044
Spain	0.1159	0.0145	0.0162	0.0026	0.0029	0.0099	0.0061
Slovenia	0.0707	0.0238	0.0132	0.0066	0.0030	0.0038	0.0117
Slovakia	0.0168	0.0119	0.0489	0.0092	0.0037	0.0107	0.0447
San Marino	0.0587	0.0101	0.0113	0.0029	0.0016	0.0012	0.0030
Sweden	0.0813	0.0112	0.0077	0.0031	0.0032	0.0197	0.0731
Portugal	0.0373	0.0153	0.0214	0.0038	0.0014	0.0041	0.0296
Norway	0.0281	0.0046	0.0044	0.0023	0.0019	0.0502	0.0816
Isle of Man	0.1006	0.0244	0.0032	0.0020	0.0019	0.0034	
Liechtenstein	0.0120	0.0201	0.0165	0.0032	0.0013	0.0009	0.0000
Lithuania	0.0349	0.0121	0.0193	0.0123	0.0028	0.0024	0.0059
Latvia	0.0268	0.0152	0.0195	0.0148	0.0023	0.0022	0.0550

Croatia	0.0393	0.0182	0.0287	0.0123	0.0081	0.0131	0.0659
Czech Republic	0.0290	0.0162	0.0193	0.0070	0.0024	0.0048	0.0097
Netherlands	0.1216	0.0071	0.0072	0.0022	0.0003	0.0016	0.0000
Guernsey	0.0635	0.0000	0.0019	0.0010	0.0010	0.0024	0.0054
Finland	0.0425	0.0096	0.0067	0.0043	0.0038	0.0117	0.0402
Faroe Islands			0.0059	0.0026	0.0005		
France	0.1865	0.0142	0.0149	0.0028	0.0012	0.0016	0.0071
Germany	0.0476	0.0267	0.0213	0.0075	0.0012	0.0024	0.0060
Denmark	0.0479	0.0043	0.0095	0.0015	0.0013	0.0060	0.0298
Belgium	0.1574	0.0171	0.0131	0.0030	0.0017	0.0033	0.0077
Austria	0.0503	0.0197	0.0193	0.0060	0.0010	0.0013	0.0020
Andorra	0.0608	0.0035	0.0107	0.0014	0.0006	0.0013	0.0056
Estonia	0.0316	0.0064	0.0101	0.0060	0.0019	0.0091	0.0254
Ireland	0.0673	0.0086	0.0152	0.0017	0.0021	0.0089	0.0196
China	0.0545	0.0122	0.0322	0.0157	0.0034	0.0003	0.0059
Jordan	0.0086	0.0130	0.0129	0.0093	0.0017	-0.0004	0.0074
Iran	0.0474	0.0447	0.0147	0.0158	0.0094	0.0102	0.0195
Turkey	0.0259	0.0078	0.0090	0.0080	0.0030	0.0013	0.0066
Thailand	0.0183	0.0008	0.0078	0.0100	0.0039	0.0151	0.0068
Malaysia	0.0140	0.0034	0.0072	0.0131	0.0024	0.0024	0.0078
Lebanon	0.0195	0.0080	0.0175	0.0069	0.0035	0.0025	0.0094
Kazakhstan	0.0062	0.2350	0.0114	0.0177	0.0024	0.0004	0.0136
South Africa	0.0343	0.0258	0.0351	0.0206	0.0196	0.0142	0.0011
Namibia	0.0000	0.0088	0.0200	0.0362	0.0242	0.0043	0.0092
Mauritius	0.0293	0.0000	0.0105	0.0086	0.0015	0.0006	0.0007
Gabon	0.0078	0.0053	0.0066	0.0077	0.0028	0.0010	0.0000

Equatorial Guinea	0.0116	0.0259	0.0109	0.0109	0.0034	0.0000	0.0000
Botswana	0.0057	0.0029	0.0196	0.0086	0.0030	0.0072	0.0050
Jamaica	0.0144	0.0241	0.0205	0.0323	0.0133	0.0335	0.0364
Saint Vincent	0.0000	0.0000	0.0057	0.0190	0.0094	0.0144	0.0794
Saint Lucia	0.0000	0.0150	0.0160	0.0258	0.0063	0.0105	0.0000
Mexico	0.1483	0.0870	0.0894	0.0427	0.0111	0.0042	0.0076
Peru	0.1323	0.0767	0.0954	0.0438	0.0082	0.0056	0.0418
Grenada	0.0000	0.0095	0.0000	0.0337	0.0026	0.0043	0.0000
Costa Rica	0.0048	0.0131	0.0126	0.0131	0.0031	0.0025	0.0041
Colombia	0.0338	0.0259	0.0244	0.0258	0.0100	0.0114	0.0290
French Guiana	0.0040	0.0060	0.0051	0.0092	0.0016	0.0011	0.0000
Puerto Rico	0.0817	0.0191	0.0152	0.0052	0.0026	0.0034	0.0057
Brazil	0.0429	0.0217	0.0295	0.0274	0.0052	0.0056	0.0065
Panama	0.0191	0.0161	0.0159	0.0100	0.0022	0.0021	0.0045
Barbados	0.0722	0.0000	0.0108	0.0088	0.0038	0.0044	0.0086
Antigua and Barbuda	0.0462	0.0215	0.0335	0.0254	0.0051	0.0104	
Argentina	0.0475	0.0279	0.0195	0.0095	0.0032	0.0020	0.0023
Serbia	0.0194	0.0090	0.0102	0.0097	0.0047	0.0033	0.0062
Romania	0.0615	0.0232	0.0398	0.0346	0.0063	0.0042	0.0081
Montenegro	0.0241	0.0141	0.0177	0.0116	0.0046	0.0014	0.0037
Russia	0.0144	0.0190	0.0332	0.0348	0.0091	0.0037	0.0042
Poland	0.0423	0.0215	0.0293	0.0179	0.0102	0.0060	0.0064
Bulgaria	0.0462	0.0371	0.0478	0.0398	0.0149	0.0071	0.0184
Belarus	0.0063	0.0079	0.0077	0.0086	0.0050	0.0125	
Vietnam	0.0000	0.0318	0.0031	0.0184	0.0012	0.0001	0.0040
Indonesia	0.0510	0.0280	0.0256	0.0208	-0.0735	0.0062	0.0257

India	0.0298	0.0136	0.0124	0.0185	0.0051	0.0046	0.0121
Iraq	0.0390	0.0200	0.0058	0.0093	0.0043	0.0011	0.0107
Armenia	0.1736	-0.0120	0.0257	0.0289	0.0083	0.0038	0.0203
Uzbekistan	0.0029	0.0086	0.0037	0.0084	0.0036	0.0000	0.0067
Sri Lanka	0.0054	0.0047	0.0130	0.0364	0.0204	0.0382	0.0249
Myanmar	0.0201	0.0215	0.0192	0.0429	0.0019	0.0028	0.0316
Bangladesh	0.0127	0.0155	0.0174	0.0202	0.0028	0.0047	0.0148
Mongolia	0.0000	0.0010	0.0042	0.0026	0.0006	0.0002	0.0023
Laos	0.0000	0.0000	0.0014	0.0034	0.0030	0.0000	0.0032
Cambodia	0.0000	0.0000	0.0115	0.0348	0.0028	0.0000	0.0224
Kyrgyzstan	0.0087	0.0174	0.0147	0.0134	0.0117	0.0000	0.0149
Georgia	0.0162	0.0110	0.0203	0.0145	0.0042	0.0007	0.0100
Philippines	0.0336	0.0184	0.0163	0.0188	0.0106	0.0133	0.0165
Bhutan	0.0000	0.0000	0.0007	0.0036	0.0003	0.0000	0.0004
Palestine	0.0033	0.0098	0.0123	0.0086	0.0039	0.0011	0.0086
Pakistan	0.0206	0.0214	0.0255	0.0196	0.0061	0.0061	0.0197
Azerbaijan	0.0121	0.0119	0.0201	0.0120	0.0077	0.0088	0.0124
Tunisia	0.0426	0.0336	0.0369	0.0344	0.0096	0.0062	0.0000
Senegal	0.0000	0.0328	0.0249	0.0228	0.0067	0.0000	0.0411
Nigeria	0.0228	0.0115	0.0133	0.0123	0.0072	0.0012	0.0000
Morocco	0.0183	0.0168	0.0205	0.0129	0.0047	0.0047	
Libya	0.0287	0.0145	0.0186	0.0128	0.0064	0.0014	0.0000
Lesotho	0.0000	0.0251	0.0301	0.0187	0.0066	0.0139	
Kenya	0.0233	0.0169	0.0224	0.0157	0.0071	0.0041	0.0000
Cote d'Ivoire	0.0073	0.0054	0.0069	0.0200	0.0061	0.0056	0.0083
Cameroon	0.0241	0.0092	0.0178	0.0184	0.0074	0.0090	0.0059

Ghana				0.0100	0.0075	0.0020	0.0021
Republic of Guinea	0.0058	0.0060	0.0079	0.0253	0.0108	0.0252	0.0000
Cape Verde	0.0103	0.0094	0.0085	0.0088	0.0026	0.0026	0.0167
Benin	0.0175	0.0112	0.0132	0.0034	0.0009	0.0000	0.0000
Angola	0.0658	0.0217	0.0346	0.0325	0.0126	0.0012	0.0000
Egypt	0.0430	0.0673	0.0542	0.0538	0.0232	0.0554	0.0516
Fiji	0.0000	0.0645	0.0043	0.0137	0.0142	0.0049	0.0122
Papua New Guinea	0.0000	0.0117	0.0100	0.0219	0.0084	0.0035	0.0081
Suriname	0.0000	0.0000	0.0000	0.0000	0.0000	0.0000	0.0000
El Salvador	0.0266	0.0292	0.0320	0.0337	0.0067	0.0028	
Nicaragua	0.0367	0.0323	0.0141	0.0039	0.0222	0.0044	0.0000
Honduras	0.0265	0.0256	0.0273	0.0295	0.0099	0.0043	0.0061
Guyana	0.0522	0.0250	0.0222	0.0302	0.0071	0.0072	0.0117
Cuba	0.0368	0.0063	0.0064	0.0091	0.0015	0.0002	0.0000
Ecuador	0.0809	0.0611	0.0306	0.1373	0.0057	0.0017	0.6964
Belize	0.0833	0.0226	0.0339	0.0142	0.0026	0.0014	0.0000
Bolivia	0.0322	0.0648	0.0269	0.0184	0.0071	0.0015	0.0021
Paraguay	0.0000	0.0000	0.0000	0.0000	0.0000	0.0000	0.0000
Ukraine	0.0261	0.0172	0.0286	0.0304	0.0094	0.0063	0.0080
Georgia	0.0162	0.0110	0.0203	0.0149	0.0041	0.0007	0.0048
Bosnia and Herzegovina	0.0425	0.0362	0.0595	0.0560	0.0156	0.0191	0.0633
Azerbaijan	0.0121	0.0119	0.0201	0.0120	0.0078	0.0088	0.0510
Albania	0.0229	0.0204	0.0171	0.0099	0.0041	0.0018	0.0064
Yemen	0.2695	0.3155	0.1557	0.1942	0.0972	0.0826	0.1817
Syria	0.0335	0.0628	0.0827	0.0414	0.0442	0.0087	0.0563

Tajikistan	0.0088	0.0049	0.0042	0.0095	0.0000		0.0070
Nepal	0.0022	0.2171	0.0104	0.0129	0.0024	0.0031	0.0122
Afghanistan	0.0235	0.0694	0.0404	0.0631	0.0152	0.0050	0.0419
Central African Republic	0.0108	0.0108	0.0086	0.0022	0.0054	0.0000	0.0000
Chad	0.0878	0.0239	0.0388	0.0133	0.0052	0.0044	0.0000
Zambia	0.0151	0.0192	0.0133	0.0164	0.0036	0.0023	0.0036
Uganda	0.0000	0.0089	0.0162	0.0363	0.0134	0.0033	
Tanzania	0.0413		0.0345	0.0239	0.0200	0.0006	0.0017
Somalia	0.0310	0.0221	0.0560	0.0649	0.0086	0.0000	0.0000
Eswatini	0.0138	0.0208	0.0469	0.0133	0.0161	0.0065	0.0099
Sierra Leone	0.0421	0.0132	0.0190	0.0168	0.0028	0.0000	0.0000
Niger	0.0847	0.0047	0.0545	0.0432	0.0215	0.0074	0.0526
South Sudan	0.0194	0.0163	0.0056	-0.0079	0.0010	0.0000	
Mozambique	0.0057	0.0091	0.0122	0.0104	0.0050	0.0062	0.0044
Republic of the Congo	0.0509	0.0219	0.0228	0.0077	0.0128	0.0233	0.0026
Mauritania	0.0651	0.0064	0.0392	0.0184	0.0065	0.0039	0.0000
Mali	0.0530	0.0317	0.0388	0.0213	0.0074	0.0043	0.0000
Malawi	0.0131	0.0330	0.0333	0.0303	0.0236	0.0231	0.0025
Madagascar	0.0094	0.0155	0.0251	0.0146	0.0246	0.0084	0.0169
Rwanda	0.0020	0.0116	0.0112	0.0125	0.0057	0.0042	0.0047
Liberia	0.0500	0.0466	0.0455	0.0399	0.0076	0.0000	0.0000
Comoros	0.0314	0.0042	0.0416	0.0043	0.0018	0.0000	0.0000
Zimbabwe	0.0192	0.0267	0.0398	0.0198	0.0127	0.0182	0.0099
Guinea-Bissau	0.0145	0.0263	0.0157	0.0306	0.0116	0.0087	0.0000
Gambia	0.0000	0.0331	0.0245	0.0396	0.0120	0.0121	0.0000

Eritrea	0.0000	0.0009	0.0045	0.0254	0.0154	0.0000	
Togo	0.0218	0.0182	0.0069	0.0073	0.0037	0.0077	0.0000
Burundi	0.0059	0.0015	0.0014	0.0002	0.0001	0.0000	0.0000
Ethiopia	0.0205	0.0152	0.0160	0.0186	0.0085	0.0038	0.0004
Vanuatu		0.0000	0.0000	0.0000	0.0012	0.0000	
Solomon Islands		0.0000	0.0000	0.0000	0.0071	0.0000	
Venezuela	0.0083	0.0091	0.0131	0.0129	0.0050	0.0041	0.0117
Haiti	0.0178	0.0318	0.0239	0.0445	0.0116	0.0100	0.0000

Data source: World Health Organization
